# A systematic review and meta-analysis expounding the relationship between methylene tetrahydrofolate reductase gene polymorphism and the risk of intracerebral hemorrhage among populations

**DOI:** 10.3389/fgene.2022.829672

**Published:** 2022-08-03

**Authors:** Xue-Lun Zou, Tian-Xing Yao, Lu Deng, Lei Chen, Ye Li, Le Zhang

**Affiliations:** ^1^ Department of Neurology, Xiangya Hospital, Central South University, Changsha, China; ^2^ National Clinical Research Center for Geriatric Disorders, Xiangya Hospital, Central South University, Changsha, China; ^3^ Multi-Modal Monitoring Technology for Severe Cerebrovascular Disease of Human Engineering Research Center, Xiangya Hospital, Central South University, Changsha, China

**Keywords:** methylenetetrahydrofolate reductase, C677T, A1298C, gene polymorphism, intracerebral hemorrhage, meta-analysis

## Abstract

**Background:** The relationship between methylenetetrahydrofolate reductase (MTHFR) gene C677T and A1298C polymorphism with the risk of intracerebral hemorrhage (ICH) has remained to be controversial in recent years. This meta-analysis is aimed to confirm the association of these.

**Methods:** Systematically searching the related studies from the PubMed, Embase, Cochrane Library, China national knowledge internet database from 1 January 1990 to 1 June 2022. The odd ratio (ORs) and 95% confidence interval (CIs) of gene-disease correlation in various gene models were calculated by fixed or random effect model of meta-analysis. We included 20 case-control studies in this meta-analysis with a total of 1,989 ICH patients and 4,032 health controls originated from Asian, Caucasian, and African populations.

**Results:** The statistical analysis demonstrated the association of MTHFR C677T gene polymorphism with ICH in allele model [OR_T VS. C_ = 1.20 (95%CI: 1.06–1.36)]; homozygote model [OR _TT VS. CC_ = 1.50 (95%CI: 1.20–1.88)]; dominant model [OR _CT+ TT VS. CC_ = 1.23 (95%CI: 1.03–1.48)] and recessive model [OR_TT VS. CT+CC_ = 1.37 (95%CI: 1.17–1.60)]. Besides, we also found the relationship of MTHFR C677T gene polymorphism with Asian in four comparison model (OR_T VS. C_ = 1.19.95%CI:1.09–1.37, OR_TT VS. CC_ = 1.46.95%CI: 1.15–1.85, OR _CT+ TT VS. CC_ = 1.25.95%CI: 1.01–1.54, OR_TT VS. CT+CC_ = 1.34.95%CI: 1.54–1.17) and Caucasian in four comparison model (OR_T VS. C_ = 1.90.95%CI: 1.22–2.97, OR_TT VS. CC_ = 2.67.95%CI: 1.42–5.00, OR _CT+ TT VS. CC_ = 1.56.95%CI: 1.05–2.32, OR_TT VS. CT+CC_ = 2.25.95%CI: 1.46–4.00). But no statistically significant correlation between A1298C polymorphism and the occurrence of ICH was detected in four studies.

**Conclusion:** MTHFR C677T gene polymorphism increases the risk of ICH in Asian and Caucasian populations but has no impact on the incidence in African communities. More importantly, the risk of ICH increases in TT genotype individuals in comparison to CT and CC genotype individuals in Asian and Caucasian populations.

## Introduction

ICH is a common acute cerebrovascular disease with much higher mortality rate than that of ischemic stroke (van Asch et al., 2010; Krishnamurthi et al., 2013; Krishnamurthi et al., 2020), accounted for 10–30% of strokes with incidence about 24/100,000 people per year (van Asch et al., 2010; Krishnamurthi et al., 2020) and prevalence about 300/100,000 people per year (Krishnamurthi et al., 2020). ICH is a serious threat to physical and mental health which brings huge economic burden to healthcare systems across human societies (Feigin et al., 2009; Krishnamurthi et al., 2013). Generally accepted risk factors for ICH include hypertension, use of anticoagulants or antiplatelet agents, cerebral arteriovenous malformations, cerebral amyloidosis, smoking, excessive drinking, and other environmental and generic factors (An et al., 2017). Commensurate with its implication in health and economy, more and more studies on the occurrence mechanism of ICH have shown that genes play a very important role in the pathogenesis of ICH (Chen et al., 2018; Wahab et al., 2019). However, the effect of gene polymorphism on ICH remains controversial.

MTHFR is an important enzyme in the regulation of plasma homocysteine level. Under normal physiological conditions, MTHFR catalyzes the reduction of 5, 10-methylene tetrahydrofolic acid to 5-methyl tetrahydrofolic acid and the resulting 5-methylenetetrahydrofolate is a source of methyl for the conversion of homocysteine to methionine (Goyette et al., 1994; Ogino and Wi lson, 2003). The encoded gene of MTHFR is located at 1p36.3. MTHFR C677T gene polymorphism causes valine to be replaced by alanine, leading to the decrease of MTHFR activity and the increase of plasma homocysteine concentration (Munshi et al., 2008; Zhu et al., 2015; Chen et al., 2018). High plasma homocysteine concentration in humans contributes to accelerated atherosclerosis, as well as excessive inflammation, long-term endothelial pressure, and increased plaque rupture, all of which increase susceptibility to ICH (Lai and Kan, 2015; Vacek et al., 2015; Zhou et al., 2018). In addition, previous studies have indicated that hyperhomocysteinemia is a risk factor for coronary artery disease, peripheral vascular disease, venous thrombosis and other vascular diseases (Veeranna et al., 2011; Vacek et al., 2015; Zhu et al., 2015; Zhou et al., 2018). In addition to C677T, A1298C is another common gene polymorphism in MTHFR with locus at rs1801131. The main pathological change of exon seven is the replacement of glutamate by alanine, which leads to the decrease of MTHFR activity in human body. In comparison to the C677T gene polymorphism, the MTHFR activity resulting from A1298C polymorphism is relatively higher (Viel et al., 1997; Weisberg et al., 1998).

Previously, some meta-analyses explored the relationship between MTHFR C677T or A1298C gene polymorphism and ischemic stroke, hemorrhagic stroke or coronary artery disease (Lv et al., 2013; Luo et al., 2018; Wang et al., 2021). However, there are only few meta-analyses to explore the relationship between MTHFR C677T and A1298C gene polymorphisms and ICH. Moreover, with the continuous emergence of emerging studies, there are some disputes between MTHFR C677T gene polymorphism and ICH. Recent studies in Morocco (Abidi et al., 2018), India (Somarajan et al., 2011; Sagar et al., 2018) and other countries reported contradictive results that the genetic MTHFR C677T polymorphism is not related to ICH. Interestingly, a Zambian study showed no C677TTT genotype in their population (Atadzhanov et al., 2013). Related to C677T polymorphism, significant association between the A1298C polymorphism of MTFHR and the risk of ischemic stroke in Asian population (Kang et al., 2014; Zhang et al., 2014; Kumar et al., 2020) has also been reported although no correlation studies on the susceptibility of A1298C polymorphism to ICH has been published. Therefore, we included emerging studies to update the relationship between MTHFR C677T gene polymorphism and intracerebral hemorrhage, and analyzed the impact of MTHFR C677T gene polymorphism on intracerebral hemorrhage among different populations and different regions. Besides, we also explore the relationship between MTHFR A1298C gene polymorphism with intracerebral hemorrhage.

## Methods

### Database search

This systematic review and meta-analysis was conducted following the Preferred Reporting Items for Systematic Reviews and Meta-analysis (PRISMA) statement guidelines. We systematically searched the studies published in PubMed, Embase, Cochrane Library, China national knowledge internet (CNKI) and other databases from 1 January 1990 to 1 June 2022 regarding the correlation between MTHFR C677T and A1298C polymorphism and ICH. The retrieval strategies: “methylene tetrahydrofolate reductase” or “methyl tetrahydrofolate reductase” or “MTHFR” and “polymorphism” or “mutation” or “genotype” or “A1298C” or “C677T” and “cerebral hemorrhage” or “intracerebral hemorrhage “or “hemorrhagic stroke” or “ICH” or “cerebrovascular disease” were executed for the search. The research population is limited to “human”. And the publication language is only allowed to include Chinese and English. Moreover, manual searches of the reference lists of retrieved study, review articles, and previous meta-analyses were performed to collect more relevant studies that were omitted during electronic database retrieval. Our review of literature inclusion consists of three main steps, first on the title of the study, then on the abstract, and finally on the complete text. If there is a dispute, discuss it in depth. As all data in this study are from published studies, no additional ethical approval is required.

### Eligibility criteria

Inclusion criteria: 1) a case-control study investigating the correlation between MTHFR C677T or A1298C gene polymorphism and ICH; 2) ICH was clinically confirmed by clinical and computed tomography or magnetic resonance imaging scans; 3) Genotype frequencies are available to estimate the odds ratio of the 95% confidence interval. Exclusion criteria were: 1) Duplicate publications with overlapping topics in the same study; 2) No available data were reported; 3) low-quality studies with a quality score (Newcastle-Ottawa Scale) below 4.

### Data extraction

According to the above inclusion and exclusion criteria, two investigators independently extracted data from the included literature. The extracted information includes the first author’s name, published journal, published year, country, race, an average age of population, number of case group and control group, genotyping method, A1298C and C677T genotype distribution and allele frequency. The allele frequency distribution was calculated by Hardy Weinberg equilibrium (HWE) (Wang and Shete, 2017) in studies which did not provide allele frequency information.

### Quality assessment

The quality of the included study was evaluated by Newcastle-Ottawa Scale (Stang, 2010) and was assessed from three aspects: population selection, comparability between groups, and measurement of exposure factors. The score ranged from 0 to 9, a score of 0–3 is considered as in poor quality, and a score of seven or above is considered as in high quality. Investigators strictly control the quality of articles included.

### Statistical analysis

We assessed the association between the allelic model (T versus C), homozygous model (TT versus CC), heterozygous model (CT versus CC), recessive model (TT versus TC + CC), and dominant model (TC + TT versus CC) alleles of MTHFR C677T and A1298C and the hazard of ICH. Using a fixed effect model (Mantel-Haenszel method) or random effect model (DerSimonian and Laird’s method) to calculate the odds ratio (ORs) of 95%CI (Mantel and Haenszel, 1959; DerSimonian, 1996). Heterogeneity between studies was compared using Cochran’s Q statistics and I^2^ measures (Higgins and Thompson, 2002; Higgins et al., 2003). I^2^ was used to assess the degree of heterogeneity between the included studies, in which 0–25% indicated no heterogeneity, a higher value indicated increased heterogeneity, 25–50% was considered as low, 50–75% medium, and 75–100% high. If the heterogeneity is high, we will conduct subgroup analysis and meta regression to analyze the source of heterogeneity.

In order to verify the reliability of the results of the meta-analysis, sensitivity analysis was performed by case-by-case exclusion (Tobias, 1999). Furthermore, we conducted HWE in the control group and observed changes in sensitivity by excluding studies that did not conform to HWE. In terms of publication bias, the Begg funnel graph was used for evaluation and the Egger graph for verification (Egger et al., 1997). All statistical analyses were performed with STATA 14.0, and all *p* values were bilateral. When *p* < 0.05, it was considered statistically significant.

## Results

### Literature search

**FIGURE 1:**
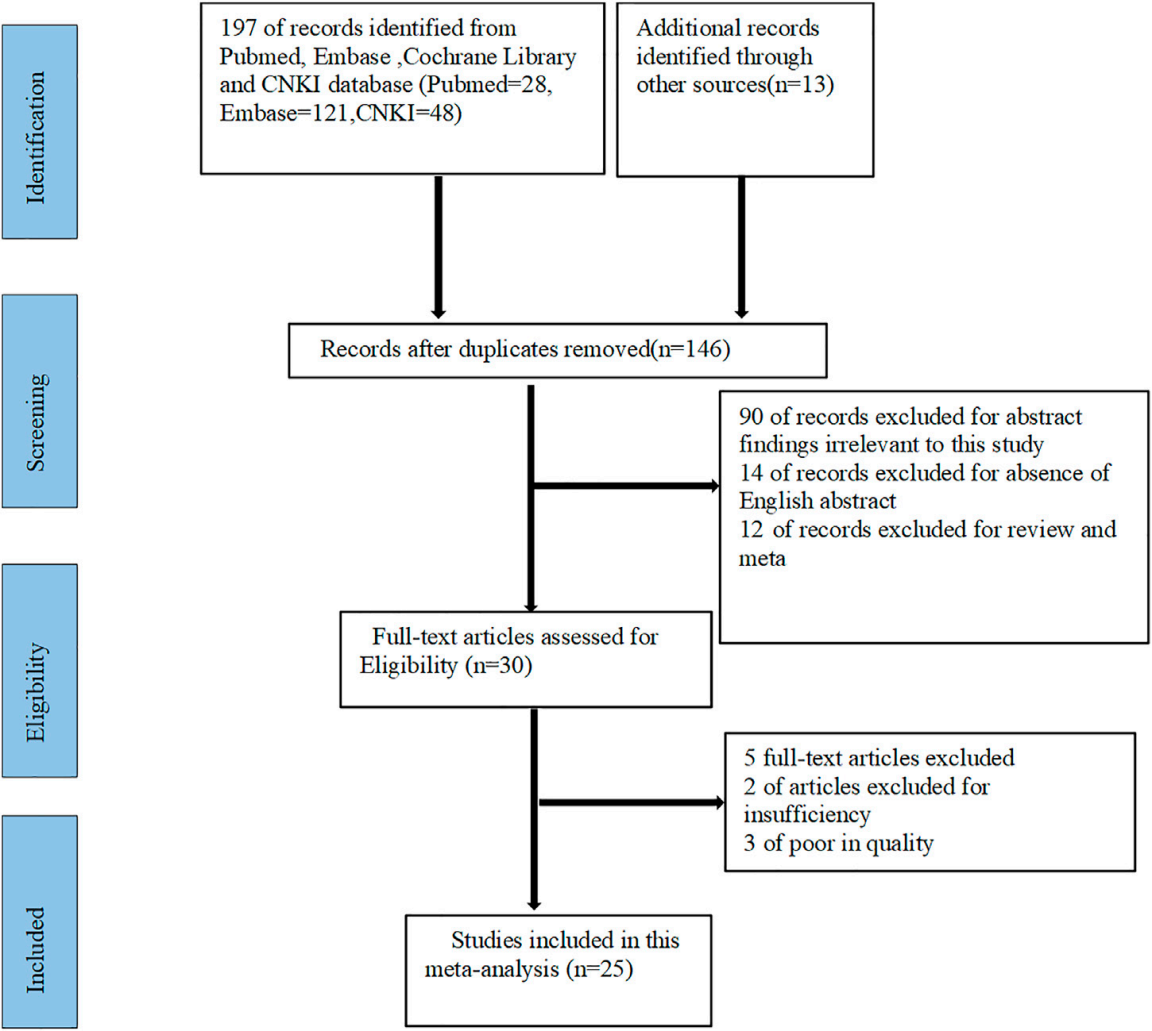
Flow diagram for the selection of studies in this meta-analysis. CNKI: China national knowledge internet.

146 studies were preliminarily searched in PubMed, Embase, Cochrane Library and CNKI databases. After screening, we excluded 90 studies that were not strongly relevant to this study, 14 studies that did not have abstracts in English, and 12 meta-analyses and reviews. Then thirty studies were left. Furthermore, we conducted an in-depth review of the full text of the 30 studies, excluding five of them with insufficient information or low quality. Finally, 25 studies were included for this meta-analysis (as showed in Figure 1).

### Study characteristics

The basic features of the 25 case-control studies (Nakata et al., 1998; Zheng et al., 2000; Yingdong et al., 2002; Li et al., 2003; Zhang et al., 2004; Yan et al., 2004; Fang and Wu, 2004; Ye et al., 2004; Żur-Wyrozumska et al., 2017; Fang et al., 2005; Dikmen et al., 2006; Sazci et al., 2006; Hu et al., 2007; Shen et al., 2007; Zhang et al., 2008; Ruigrok et al., 2010; Hultdin et al., 2011; Somarajan et al., 2011; Atadzhanov et al., 2013; Rui-Rui et al., 2013; Das et al., 2015; Shao et al., 2016; Hu et al., 2016; Abidi et al., 2018; Jiang et al., 2018) included are shown in Table 1. The incorporated studies ranged from 1998 to 2018, involving mainly Asian populations and some European and African communities. Three different races were involved in these 25 selected studies: two (Atadzhanov et al., 2013; Abidi et al., 2018) African, three (Dikmen et al., 2006; Sazci et al., 2006; Hultdin et al., 2011) Caucasian and the remaining 17 (Nakata et al., 1998; Zheng et al., 2000; Yingdong et al., 2002; Zhang et al., 2004; Yan et al., 2004; Fang and Wu, 2004; Ye et al., 2004; Żur-Wyrozumska et al., 2017; Hu et al., 2007; Zhang et al., 2008; Rui-Rui et al., 2013; Shao et al., 2016; Hu et al., 2016; Jiang et al., 2018) Asian. The control population in three of the 25 studies (Li et al., 2003; Shen et al., 2007; Das et al., 2015) did not meet Hardy-Weinberg equilibrium and be excluded. One study due to high heterogeneity (Fang et al., 2005) and one study of rebleeding after secondary subarachnoid hemorrhage (Ruigrok et al., 2010) were excluded (Table 2). All the 20 included studies had moderately high-quality scores. In four of the 20 studies (Dikmen et al., 2006; Sazci et al., 2006; Hultdin et al., 2011; Abidi et al., 2018), A1298C and C677T gene polymorphism and ICH were simultaneously studied, and a total of 3,280 patients with ICH and 9,324 controls were included in this meta-analysis.

**TABLE 1:** The characteristics of all studies in the meta-analysis of the association between MTHFR gene C677T and A1298C polymorphisms with the risk of intracerebral hemorrhage. PCR-RFLP = Polymerase Chain Reaction-Restriction Fragment Length Polymorphism. SNP = Single Nucleotide Polymorphism. HWE = Hardy Weinberg Equilibrium. QS = Quality score.

Author	Year	Origin	Ethnicity	Size (Case/Control)	Mutation	Technology	HWE	QS
Nakata Y Nakata et al., 1998	1998	Japan	Asian	35/105	C677T	PCR-FRLP	Yes	5
Zheng Y Z Zheng et al., 2000	2000	China	Asian	30/122	C677T	PCR-FRLP	Yes	5
Zhao Y Żur-Wyrozumska et al., 2017	2001	China	Asian	202/190	C677T	PCR	Yes	4
Fang L Fang and Wu, (2004)	2004	China	Asian	27/96	C677T	PCR-FRLP	Yes	4
Zhang J Zhang et al., 2004	2004	China	Asian	94/100	C677T	PCR-FRLP	Yes	5
Zhang Y Yan et al., 2004	2004	China	Asian	156/239	C677T	PCR-FRLP	Yes	4
Ye H Ye et al., 2004	2004	China	Asian	100/300	C677T	PCR-FRLP	Yes	5
Fu Y Yingdong et al., 2002	2005	China	Asian	26/29	C677T	PCR-RFLP.	Yes	4
Sazci A Sazci et al., 2006	2006	Turkey	Caucasian	28/259	C677T	PCR-RFLP	Yes	5
					A1298C			
Dikmen M Dikmen et al., 2006	2006	Turkey	Caucasian	49/55	C677T	PCR-FRLP	Yes	6
					A1298C			
Hu RL Hu et al., 2007	2007	China	Asian	32/115	C677T	Sequenom	Yes	5
Zhang Y Zhang et al., 2008	2008	China	Asian	222/282	C677T	PCR-FRLP	Yes	4
Somarajan BI Somarajan et al., 2011	2011	India	Asian	215/188	C677T	PCR	Yes	6
Hultdin J Hultdin et al., 2011	2011	Sweden	Caucasian	60/778	C677T	PCR-FRLP	Yes	7
					A1298C			
Atadzhanov M Atadzhanov et al., 2013	2013	Zambian	African	18/116	C677T	TaqMan	Yes	5
Rui-Rui L I Rui-Rui et al., 2013	2013	China	Asian	182/200	C677T	PCR-RFLP	Yes	5
Shao D (Shao et al., 2016)	2015	China	Asian	96/206	C677T	PCR-FRLP	Yes	4
Hu X Hu et al., 2016	2016	China	Asian	180/180	C677T	PCR-RFLP.	Yes	5
Abidi O Abidi et al., 2018	2018	Morocca-n	African	113/323	C677T	PCR-PFLP	Yes	6
					A1298C			
Jiang D Jiang et al., (2018)	2018	China	Asian	124/149	C677T	SNa Pshot	Yes	5

**TABLE 2:** Information for some strong correlation research not included in the data collection process.

Author	Year	Origin	Ethnicity	Mutation	HWE	Delete reason
Zhaohui Li (Li et al., 2003)	2003	China	Asian	C677T	No	HWE not satisfied
Fang X (Fang et al., 2005)	2005	Japan	Asian	C677T	Yes	Large heterogeneity
Shen C D (Shen et al., 2007)	2007	China	Asian	C677T	No	HWE not satisfied
Ruigrok YM (Ruigrok et al., 2010)	2010	Dutch	Caucasian	C677T	Yes	Secondary to SAH, does not meet the requirements
Das (Das et al., 2015)	2015	India	Asian	C677T	NO	HWE not satisfied
Wenjing Ou (Ou et al., 2014)	2014	China	Asian	C677T	Yes	Unable to obtain sufficient raw data
Cai (Wang et al., 2021)	2005	China	Asian	C677T	NO	Unable to obtain sufficient raw data
Xiao (Wang et al., 2021)	2006	China	Asian	C677T	NO	Unable to obtain sufficient raw data
Sagar R (Sagar et al., 2018)	2018	India	Asian	C677T	NA	Unable to obtain sufficient raw data

### The association of methylenetetrahydrofolate reductase C677T gene polymorphism with the risk of intracerebral hemorrhage

**FIGURE 2:**
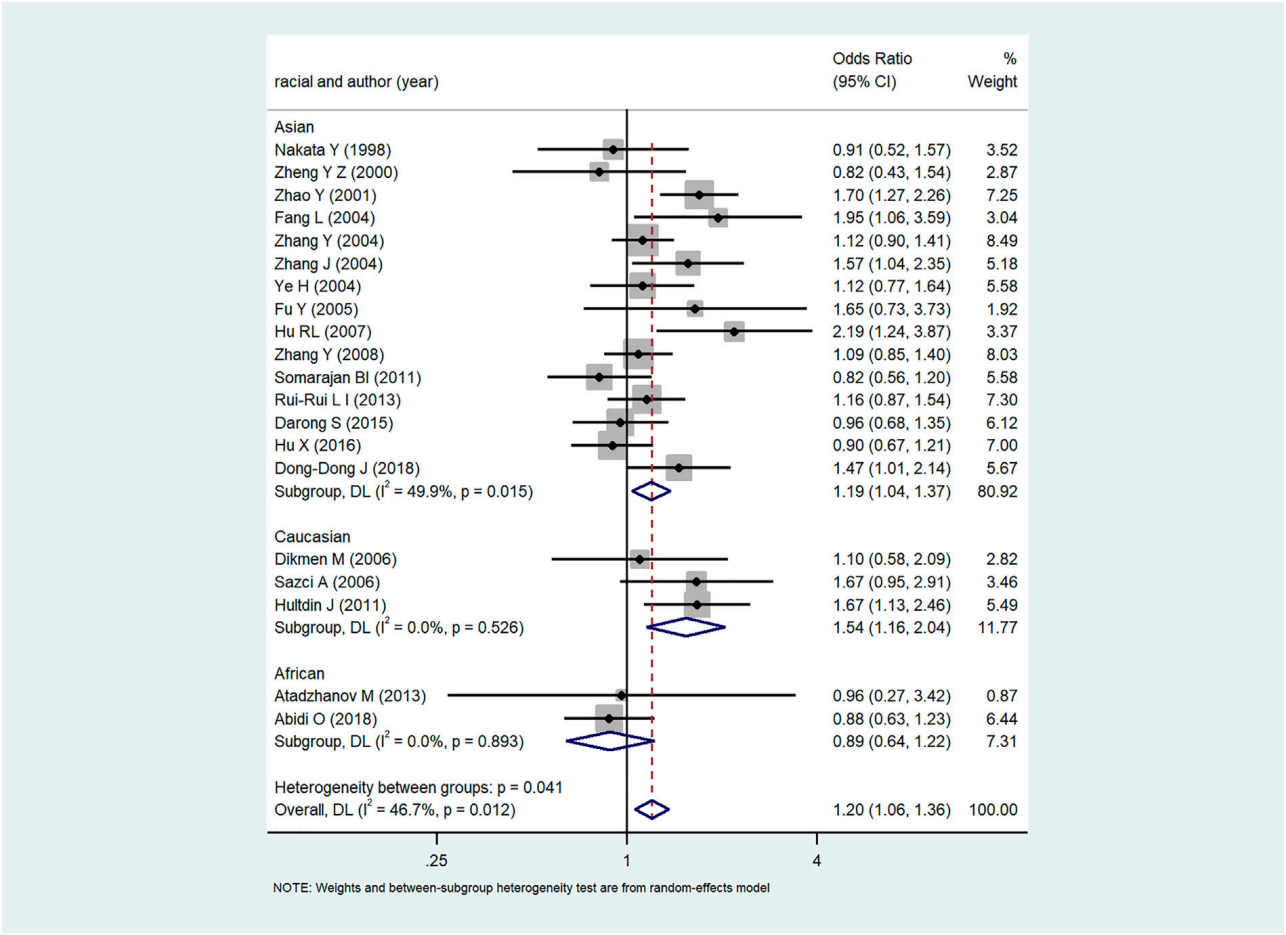
Homozygote gene model (TT VS. CC) in Asians, Caucasians, and Africans.

The forest plot of the relationship between MTHFR C667T gene polymorphism and ICH in various gene models were demonstrated in Figure 2 and Supplementary Figure S1 with the OR and *p*-values of each gene model shown in Table 3. In each gene model, a significant association was observed between MTHFR C677T gene polymorphism and the risk of ICH. The results of subgroup analysis by ethnicity varied with the risk of ICH in the study populations. MTHFR C677T gene polymorphism in the dominant model [OR _CT+ TT VS. CC_ = 1.23 (95%CI: 1.03–1.48)], allele model [OR = 1.20 (95%CI: 1.06–1.36)], homozygous model [OR = 1.50 (95%CI: 1.20–1.88)] and recessive model [OR = 1.37 (95%CI: 1.17–1.60] all revealed significant association with the risk of ICH. In addition, four comparison model (OR_T VS. C_ = 1.19.95%CI:1.09–1.37, OR_TT VS. CC_ = 1.46.95%CI: 1.15–1.85, OR _CT+ TT VS. CC_ = 1.25.95%CI: 1.01–1.54, OR_TT VS. CT+CC_ = 1.34.95%CI: 1.54–1.17) also significantly related to Asian population. Likewise, in Caucasian population, allele model [(OR = 1.90 (95%CI: 1.22–2.97)] homozygous model [OR = 2.67 (95%CI: 1.42–5.00)], recessive model [OR = 2.25 (95%CI: 1.46–4.00)] and dominant model [OR _CT+ TT VS. CC_ = 1.56.95%CI: 1.05–2.32)] showed significant association with the risk of ICH, while the association in heterozygous model [OR = 1.39 (95%CI:0.91–2.13)], were not evidenced. In African population, no substantial correlation between MTHFR C667T gene polymorphism and ICH was observed in any one of the five models with heterozygote model [OR = 0.86 (95%CI: 0.56–1.32)], dominant model [OR = 0.85 (95%CI: 0.57–1.28), allele model [OR = 0.89 (95%CI: 0.64–1.22)], homozygous model [OR = 0.82 (95%CI: 0.39–1.73)] and recessive model [OR = 0.89 (95%CI: 0.43–1.81)].

**TABLE 3:** Results of association between C677T and A1298C with intracerebral hemorrhage in this meta-analysis.

	Comparison	Number of studies	p	I2(%)	OR (95%CI)	Comparison Model
C677T overall						
	T VS C	20	0.012	46.7	1.20 (1.06–1.36)	REM
	TT VS CC	20	0.097	31.1	1.50 (1.20–1.88)	REM
	CT VS CC	20	0.256	15.9	1.09 (0.94–1.26)	REM
	CT+TT VS CC	20	0.006	49.4	1.23 (1.03–1.48)	REM
	TT VS CC+CT	20	0.398	4.80	1.37 (1.17–1.60)	REM
Asian	T VS C	15	0.015	49.9	1.19 (1.09–1.37)	REM
	TT VS CC	15	0.134	29.6	1.46 (1.15–1.85)	REM
	CT VS CC	15	0.212	21.8	1.08 (0.92–1.28)	REM
	CT+TT VS CC	15	0.04	54.9	1.25 (1.01–1.54)	REM
	TT VS CC+CT	15	0.461	0	1.34 (1.54–1.17)	REM
Caucasian	T VS C	3	0.526	0	1.90 (1.22–2.97)	REM
	TT VS CC	3	0.842	0	2.67 (1.42–5.00)	REM
	CT VS CC	3	0.352	4.20	1.39 (0.91–2.13)	REM
	CT+TT VS CC	3	0.406	0	1.56(1.05-2.32)	REM
	TT VS CC+CT	3	0.693	0	2.25 (1.46–4.00)	REM
African	T VS C	2	0.893	0	0.89 (0.64–1.22)	REM
	TT VS CC	2	…	0	0.82 (0.39–1.73)	REM
	CT VS CC	2	0.86	0	0.86 (0.56–1.32)	REM
	CT+TT VS CC	2	0.853	0	0.85 (0.57-1.28)	REM
	TT VS CC+CT	2	…	0	0.89 (0.43–1.81)	REM
A1298C						REM
	C VS A	4	0.452	0	0.86 (0.69–1.09)	FEM
	CC VS AA	4	0.084	54.9	0.72 (0.24–2.18)	REM
	AC VS AA	4	0.652	0	0.94 (0.70–1.26)	FEM
	CC+AC VS AA	4	0.810	0	0.89 (0.67–1.18)	FEM
	CC VS AC+AA	4	0.037	64.5	0.79 (0.23–2.65)	REM

REM: random effect model.

### Publication bias

**FIGURE 3:**
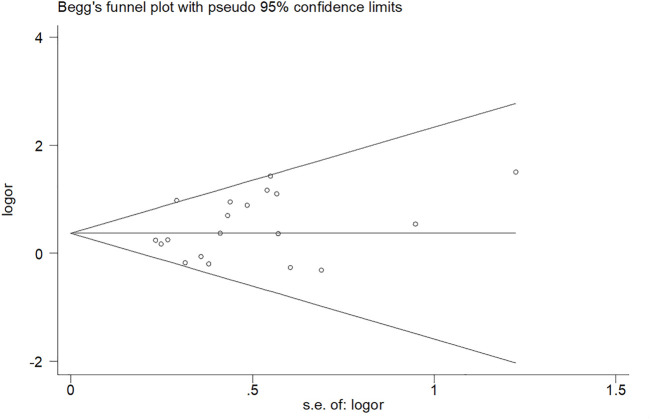
Begg’s funnel plot of the relationship between MTHFR gene (C677T) polymorphism and intracerebral hemorrhage risk.

In this paper, Begg and Egger funnel plots were applied to assess the publication bias of the 20 included studies. No significant asymmetry was observed in any gene model by either of our two researchers (Figure 3 and Supplementary Figure S2). The *p*-values of Begg and Egger’s test in all the five gene models were all over 0.05 with the dominant model (P _Begg_ = 0.098, P _Egger_ = 0.149), homozygous model (P _Begg_ = 0.294, P _Egger_ = 0.183), heterozygous model (P _Begg_ = 0.098, P _Egger_ = 0.200), allelic model (P _Begg_ = 0.381, P _Egger_ = 0.398), recessive model (P _Begg_ = 0.162, P _Egger_ = 0.264), which confirmed the absence of obvious publication bias in the 20 included studies.

### Sensitivity analyses

A sensitivity analysis of the 20 included studies was performed to assess the impact of each study on ORs inclusion by sequentially omitting individual inclusion studies. No significant change in OR value was observed in the process of removing the study one by one. Therefore, overall sensitivity analysis reinforced that the results of this meta-analysis are statistically reliable. The correlation of MTHFR gene C677T polymorphism with the risk of ICH in this meta-analysis was validated by the following meta regression analysis of the selected 21 studies, i.e., published year (*p* = 0.086), the quality score (*p* = 0.795), race [Caucasian populations (*p* = 0.100), African populations (*p* = 0.243), the Asian population (*p* = 0.931)] as a covariate meta regression analysis, with all the *p*-values above 0.05 which indicated no statistically significant. And the subgroup analysis did not find obvious source of heterogeneity.

### The association of methylenetetrahydrofolate reductase A1298C gene polymorphism with the risk of intracerebral hemorrhage

At present, there are still few studies on the relationship between MTHFR A1298C gene polymorphism and the risk of ICH only in Caucasian and African populations. Therefore, only four related studies (Dikmen et al., 2006; Sazci et al., 2006; Hultdin et al., 2011; Abidi et al., 2018) were included in the current paper. The meta-analysis results of the four studies were shown in Table 3 and Supplementary Figure S3 with corresponding OR and 95%CI for the five gene models as follows—heterozygous model [OR = 0.94 (95%CI: 0.7–1.26)], dominant model [OR = 0.87 (95%CI: 0.67–1.18), homozygous model [OR = 0.72 (95%CI: 0.24–2.18), recessive model [OR = 0.64 (95%CI: 0.34–1.2)] and allele model [OR = 0.86 (95%CI: 0.69–1.09)]. Hence, no significant correlation between MTHFR A1298C allele polymorphism and ICH was detected in any one of the five gene models in Caucasian and African communities. Furthermore, no obvious publication bias was found in Begg funnel chart (Supplementary Figure S4). Considering the absence of adequate studies, sensitivity analysis, subgroup analysis or meta-regression was not conducted.

## Discussion

In this meta-analysis, we included a total of 20 case-control studies, all 20 of which examined the correlation of the polymorphism of the MTHFR C677T gene with the risk of ICH (including 1,989 cases of ICH patients and 4,032 controls), while only four of the 20 studies interrogated the relationship of MTHFR A1298C gene polymorphism with the risk of ICH (with a total of 250 cases of ICH and 1,415 controls included). The results of the meta-analysis revealed significant association between MTHFR C677T gene polymorphism and risk of ICH under all the four genetic models in Asian populations. Funnel figure, subgroup analysis and sensitivity analysis all confirmed the reliability of the results. A subgroup analysis and a meta regression was performed to analyze the source of the heterogeneity, but no covariate was found to attribute to the source of heterogeneity.

Subgroup analysis based on ethnic classification uncovered a strong association between MTHFR C677T gene polymorphism and the risk of ICH in Asian, and Caucasians populations, while no association was found in Africans. What’s more, compared with genotype CC or, CT, TT genotype substantially increases the susceptibility to ICH with OR _TT VS. CC_ = 1.50 (95%CI: 1.20–1.88) and OR_TT VS. CT+CC_ = 1.37 (95%CI: 1.17–1.60). Considering the influence of MTHFR C677T gene polymorphism on homocysteine level, individuals with TT genotype have a higher risk of ICH which may be related to the significantly increased level of homocysteine. The revealed correlation provides a logic scientific basis for further in-depth study of gene polymorphism and molecular epidemiology of ICH.

The T-allele of methylene tetrahydrofolate reductase C677T can increase homocysteine levels in human body to a mild to moderate level (Cronin et al., 2005; Holmes et al., 2011). Artificially induced hyperhomocysteinemia mice were significantly more vulnerable to vascular inflammation, atherosclerosis, and hypercoagulability (Hofmann et al., 2001; Zhou et al., 2008; Veeranna et al., 2011; Vacek et al., 2015; Zhou et al., 2018). Likewise, MTHFR gene polymorphism also increases the risk of ICH by affecting the blood clotting function *via* hyperhomocysteinemia which accelerates the activation of coagulation factors V,X, and XII and rises the risk of arterial thrombosis and atherosclerotic cerebrovascular diseases of large and small arteries (Diaz-Arrastia, 2000; Kelly et al., 2002; Dikmen et al., 2006). Thinking on, investigations on reduction the level of homocysteine *via* intentionally ingestion of additional folic acid to mitigate the risk of ICH and coronary heart disease have been implemented. However, the clinical efficacy of folic acid on alleviation of ICH risk remains contentious (Lewis et al., 2005; Aléssio et al., 2011). Considering the common occurrence of T allele of C677T in Asian and Caucasian populations, the importance of correlation investigations for the prediction, prevention and treatment of ICH in these populations cannot be overlooked.

The current and previous studies (Gao et al., 2012; Kang et al., 2013; Zhao and Jiang, 2013; Kang et al., 2014) on the association between MTHFR gene polymorphism and the risk of ICH were mainly concentrated on C677T polymorphism in Asian populations. No research on the influence of A1298C polymorphism on the danger of ICH has been published in Asian populations. Consequently, only four studies were included in this paper involving the association of MTHFR A1298C polymorphism with ICH in Caucasian and African populations. Considering the potential correlation of A1298C gene polymorphism with the susceptibility to ICH, further studies are warranted, especially for the Asian populations with high frequency of occurrence.

Compared with the previous meta-analysis (Gao S, et al., 2012), which only focused on MTHFR C677T, this systematic review and meta-analysis analyzed the relationship between MTHFR A1298C gene polymorphism and intracerebral hemorrhage. We also included emerging studies to update the relationship between MTHFR C677T gene polymorphism and intracerebral hemorrhage, and analyzed the impact of MTHFR C677T gene polymorphism on intracerebral hemorrhage among different populations and different regions. In our study, we found that MTHFR C677T gene polymorphism increases the risk of ICH in Asian and Caucasian populations but has no impact on the incidence in African communities. More importantly, the risk of ICH increases in TT genotype individuals in comparison to CT and CC genotype individuals in Asian populations. Therefore, our study not only revealed the important role of MTHFR A1298C in the prevention and treatment of ICH, but also indicated that the populational specific strategies for ICH prevention *via* MTHFR C677T and A1298C should be considered in Asians and Caucasians instead of Africans firstly.

Due to availability of data, there are several deficiencies in this research. First of all, only a small number of studies were included, mainly on A1298C genotype, which would affect the representativeness of the results of the meta-analysis in this part. Although we searched a large number of databases, only four relevant literatures were found. Secondly, all the included studies were case-control studies, which were basically retrospective, and there was probable bias caused by partial design when comparing with prospective studies. Moreover, some gene models showed moderate to low degree of heterogeneity, but meta-regression and subgroup analysis based on publication year, ethnicity, and quality score could not designate the source of heterogeneity, which may be related to study design, genetic and environmental interaction, and the number of included patients. Finally, we have not registered this search protocol in an online database such as PROSPERO.

In summary, this meta-analysis confirmed that MTHFR C677T gene polymorphism is related to the risk of ICH, mainly in Asian populations, and that TT genotype individuals have a higher risk of ICH than CC and CT genotypes. No substantial correlation of MTHFR A1298C polymorphisms with the risk of ICH were found. The high incidence of ICH in Asian population may be related to the polymorphism of MTHFR C677T gene, which may be a predictor for the susceptibility of ICH in Asian. The detection of MTHFR C677T gene polymorphism in clinical will help to predict, prevent and reduce the pathological cases of cerebral hemorrhage in Asian population. Further studies are needed to clarify the prevention of ICH in Asian population targeting MTHFR C677T gene polymorphism.

### Main messages

1) MTHFR is an important enzyme in the regulation of plasma homocysteine level. High plasma homocysteine concentration in humans contributes to increase susceptibility to ICH.
2) MTHFR C677T gene polymorphism is a biomarker gene of ICH in Asian and Caucasian populations, which has guiding significance for the prevention of ICH.
3) The risk of ICH increases in C677TTT genotype individuals in comparison to CT and CC genotype individuals in Asian and Caucasian populations.

### Current research questions

1) Are C677T and A1298C gene polymorphisms of MTHFR associated with the risk of ICH?
2) Is there population difference in the effect of MTHFR gene polymorphism on the risk of ICH?
3) Are the different alleles of C677T and A1298C responsible for the difference in the risk of ICH in different populations? Can these allele differences become biomarkers for the prevention and treatment of ICH?
4) What is the potential mechanism of MTHFR C677T and A1298C gene polymorphisms on ICH?

## Data Availability

The raw data supporting the conclusions of this article will be made available by the authors, without undue reservation.

## Supplementary material

Supplementary Figure S1 Forest plot of the association between MTHFR C677T gene polymorphism and the risk of ICH in Asians, Caucasians, and Africans. **(A)** Allele model (T VS C) in Asians, Caucasians, and Africans. T and C are the case group and T1 and C1 are the control group. **(B)** Recessive gene model (TT VS CC+CT) in Asians, Caucasians, and Africans. where R=CC+CT, R1=CT1+CC1, R and CC are the case group, and R1 and CC1 are the control group. **(C)** Dominant gene model (TT+CT VS CC) in Asians, Caucasians, and Africans, in which TT and CC are case groups and TT1 and CC1 are control groups. **(D)** Heterozygote gene model (CT VS CC) in Asians, Caucasians, and Africans, in which CT and CC are case groups and CT1 and CC1 are control groups.

Supplementary Figure S2 Sensitivity analysis of MTHFR C677T polymorphism and risk of ICH in a dominant gene model.

Supplementary Figure S3 Forest plot of the relationship between MTHFR A1298C gene polymorphism and the risk of ICH. **(A)** Allele model (C VS A), where C and A are case groups and C1 and A1 are control groups.**(B)** Homozygous model (CC VS AA), in which CC and AA are case groups and CC1 and AA1 are control groups. **(C)** Dominant model (CC+AC VS AA), where F=CC+AC, F1=CC1+AC1.F and AA are case groups, and F1 and AA1 are control groups. **(D)** Recessive model (CCVS AA+AC), where B=AA+AC, B1=AA1+AC1.B, AA is the case group, and B1, AA1 are the control group. **(E)** Heterozygous model (AC VS AA), in which AC and AA are case groups and AC1 and AA1 are control groups.

Supplementary Figure S4 The Begg funnel plot of the relationship between MTHFR A1298C gene polymorphism and the risk of ICH.

Supplementary Table S1 Differences between other meta-analyses similar to this study.

Supplementary Table S2 Information for some strong correlation research not included in the data collection process.

## Abbreviations

MTHFR, methylenetetrahydrofolate reductase; ICH, intracerebral hemorrhage; OR, odd ratio; CI, confidence interval; CNKI, China national knowledge internet; HWE, hardy weinberg equilibrium; REM, random effect model; FEM, fixed effect model.
