# Supplementary material for: A systematic review and meta-analysis expounding the relationship between methylene tetrahydrofolate reductase gene polymorphism and the risk of intracerebral hemorrhage among populations
Source: Front Genet. 2022 Aug 3;13:829672. doi: 10.3389/fgene.2022.829672 (PMC9382188; doi:10.3389/fgene.2022.829672)
Supplement: Supplementary file 1 [file Table1.docx]

**Supplemental Table 1:** **Differences between other meta-analyses similar to this study.**

| Years | PMID | Objective | Ethnicity | Number of study |
| --- | --- | --- | --- | --- |
| 2012 | 22938732 | intracerebral hemorrhage | Asian(14) Caucasian(2) | 16 C677T |
| 2013 | 23428159 | hemorrhagic stroke | Asian(11) European(4) | 15 C677T |
| 2013 | 23184002 | hemorrhagic stroke | Asian(11) Caucasian(1) | 12 C677T |
| 2016 | 26776436 | ischemic stroke | NA | 22 C677T |
| 2018 | 30115070 | coronary artery disease | Asian  Caucasian African | 123 C677T |
| 2021 | 33247557 | intracranial hemorrhage | Asian Caucasian Mixed | 8 A1298C+ 31 C677T |
| 2013 | 24391036 | stroke | White Asian | 13 A1298C |
| 2020 | 32727306 | stroke | Asian Caucasian | 20 A1298C |
