# Supplementary material for: A systematic review and meta-analysis expounding the relationship between methylene tetrahydrofolate reductase gene polymorphism and the risk of intracerebral hemorrhage among populations
Source: Front Genet. 2022 Aug 3;13:829672. doi: 10.3389/fgene.2022.829672 (PMC9382188; doi:10.3389/fgene.2022.829672)
Supplement: Supplementary file 2 [file Datasheet1.docx]

**Supplementary Material**

**Supplementary Figure 1:** **Forest plot of the association between MTHFR C677T gene polymorphism and the risk of ICH in Asians, Caucasians, and Africans. a.** Allele model (T VS C) in Asians, Caucasians, and Africans. T and C are the case group and T1 and C1 are the control group. **b.** Recessive gene model (TT VS CC+CT) in Asians, Caucasians, and Africans. where R=CC+CT, R1=CT1+CC1, R and CC are the case group, and R1 and CC1 are the control group. **c.** Dominant gene model (TT+CT VS CC) in Asians, Caucasians, and Africans, in which TT and CC are case groups and TT1 and CC1 are control groups. **d.** Heterozygote gene model (CT VS CC) in Asians, Caucasians, and Africans, in which CT and CC are case groups and CT1 and CC1 are control groups.

**Supplementary Figure 2: Sensitivity analysis of MTHFR C677T polymorphism and risk of ICH in a dominant gene model.**

**Supplementary Figure 3**: **Forest plot of the relationship between MTHFR A1298C gene polymorphism and the risk of ICH.** **a.** Allele model (C VS A), where C and A are case groups and C1 and A1 are control groups. **b.** Homozygous model (CC VS AA), in which CC and AA are case groups and CC1 and AA1 are control groups. **c.** Dominant model (CC+AC VS AA), where F=CC+AC, F1=CC1+AC1.F and AA are case groups, and F1 and AA1 are control groups. **d.** Recessive model (CCVS AA+AC), where B=AA+AC, B1=AA1+AC1.B, AA is the case group, and B1, AA1 are the control group.

**e.** Heterozygous model (AC VS AA), in which AC and AA are case groups and AC1 and AA1 are control groups.

**Supplementary Figure 4: The Begg funnel plot of the relationship between MTHFR A1298C gene polymorphism and the risk of ICH.**


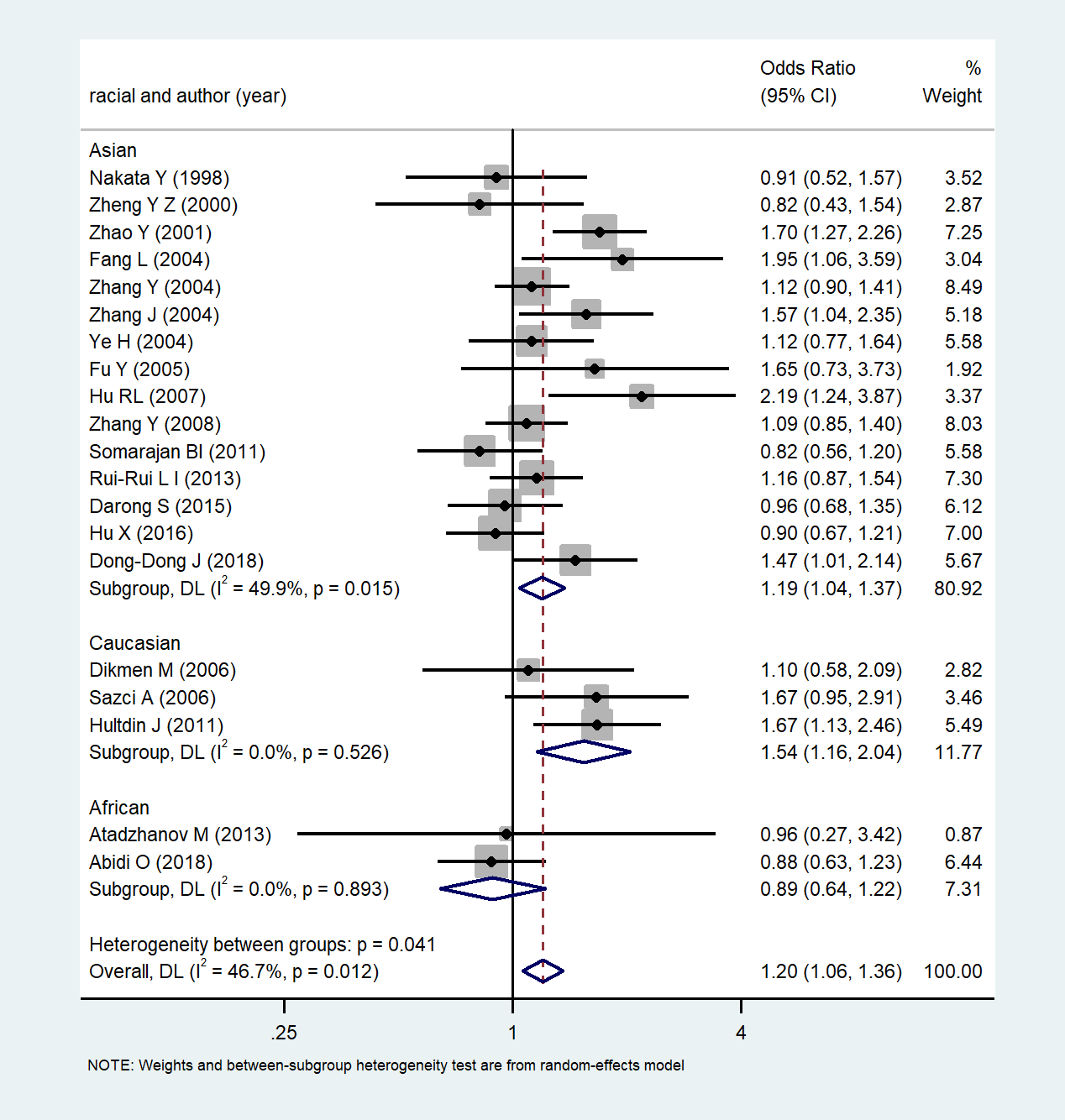


**Supplementary Figure 1(a)**


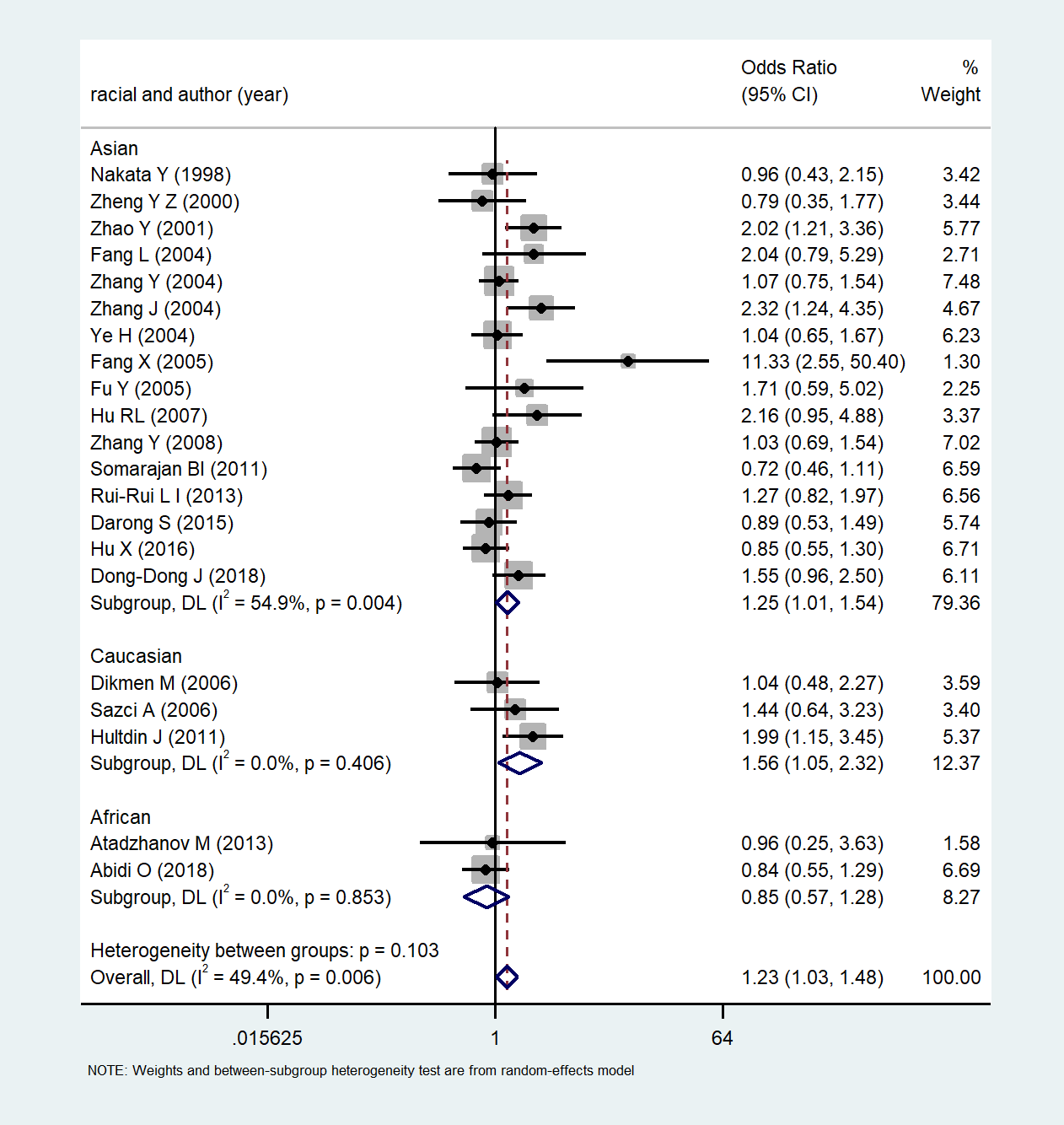


**Supplementary Figure 1 (b)**


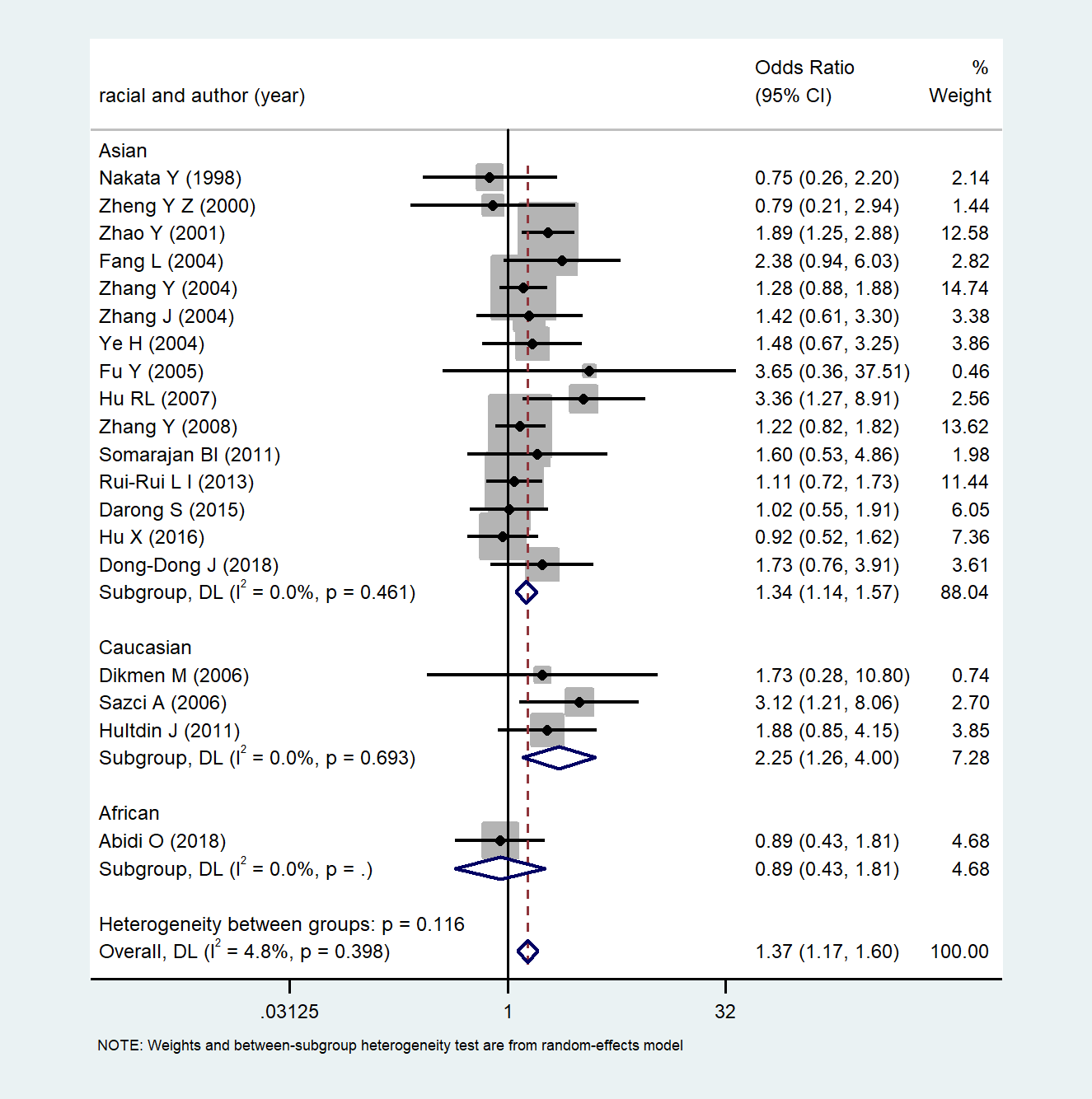


**Supplementary Figure 1(c)**


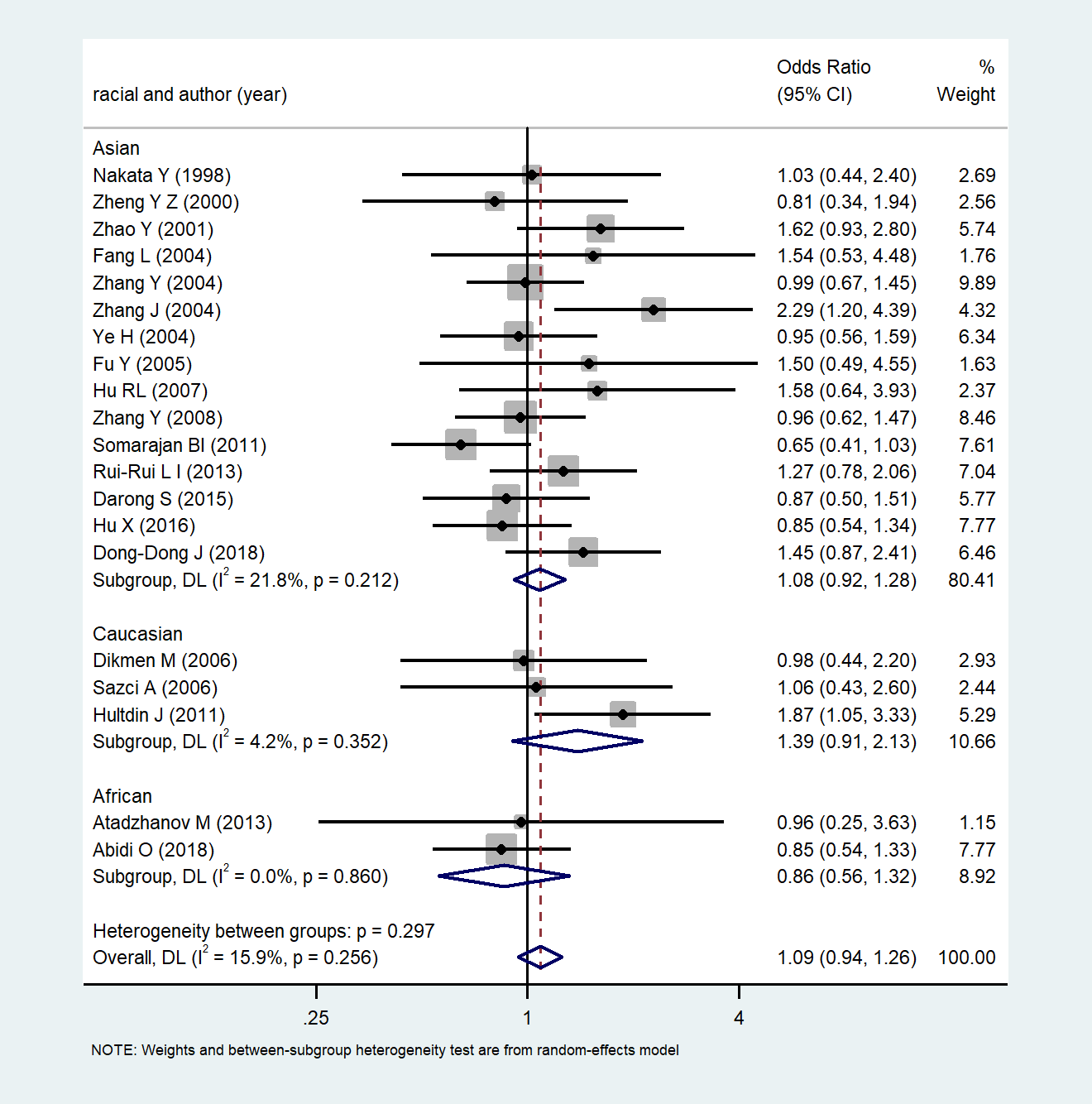


**Supplementary Figure 1(d)**

**Supplementary Figure 1:** **Forest plot of the association between MTHFR C677T gene polymorphism and the risk of ICH in Asians, Caucasians, and Africans.**

(a). Allele model (T VS C) in Asians, Caucasians, and Africans. T and C are the case group and T1 and C1 are the control group. (b) Recessive gene model (TT VS CC+CT) in Asians, Caucasians, and Africans. where R=CC+CT, R1=CT1+CC1, R and CC are the case group, and R1 and CC1 are the control group. (c)Homozygous gene model (TT VS CC) in Asians, Caucasians, and Africans, in which TT and CC are case groups and TT1 and CC1 are control groups. (d) Heterozygote gene model (CT VS CC) in Asians, Caucasians, and Africans, in which CT and CC are case groups and CT1 and CC1 are control groups.


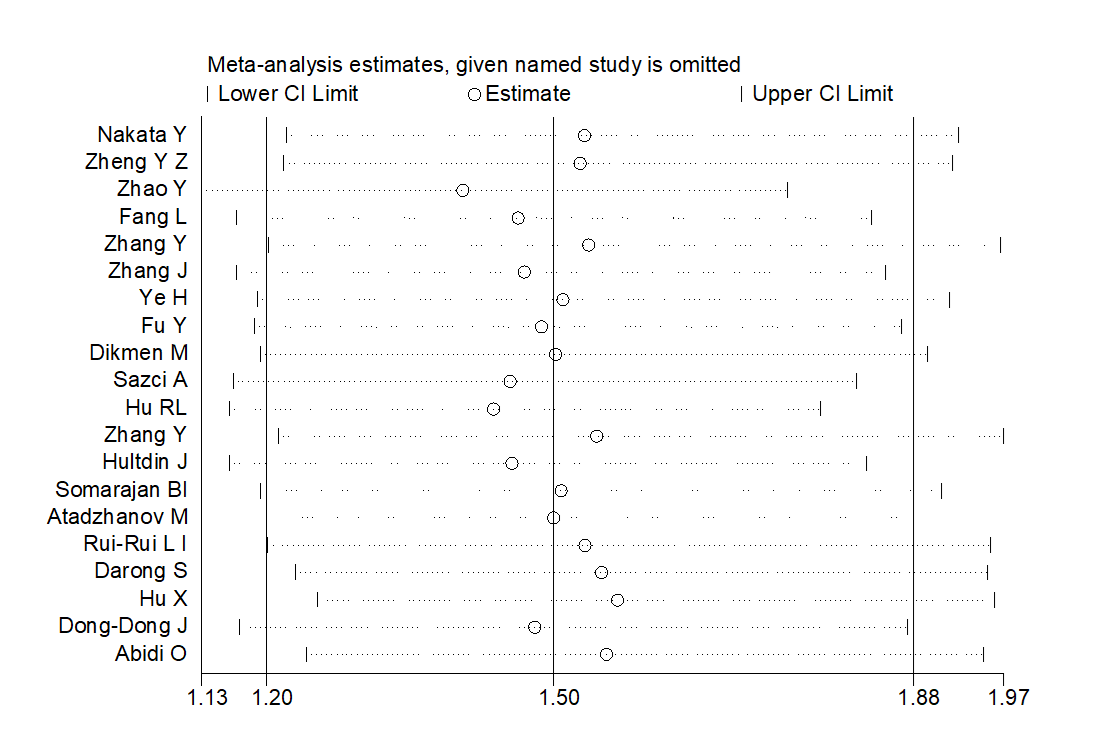


**Supplementary Figure 2: Sensitivity analysis of MTHFR C677T polymorphism and risk of ICH in a dominant gene model.**


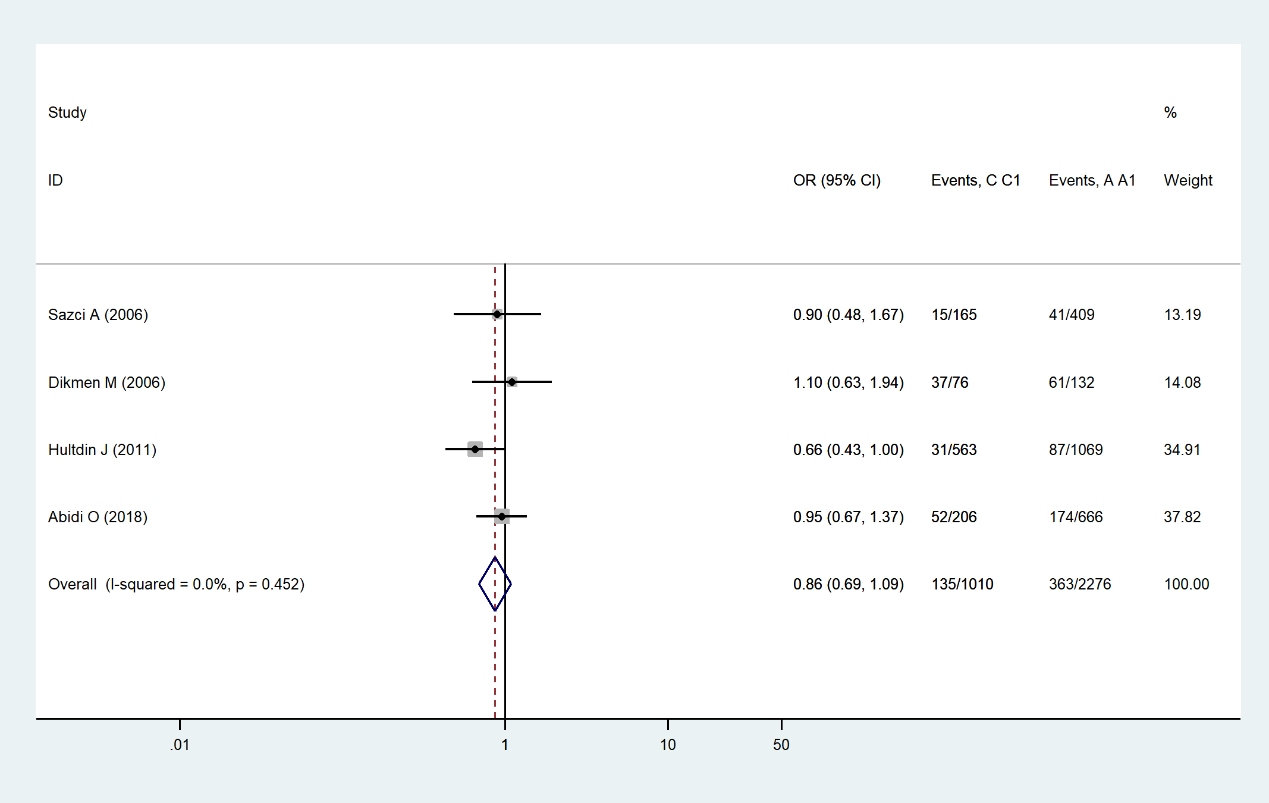


**Supplementary Figure 3(a)**


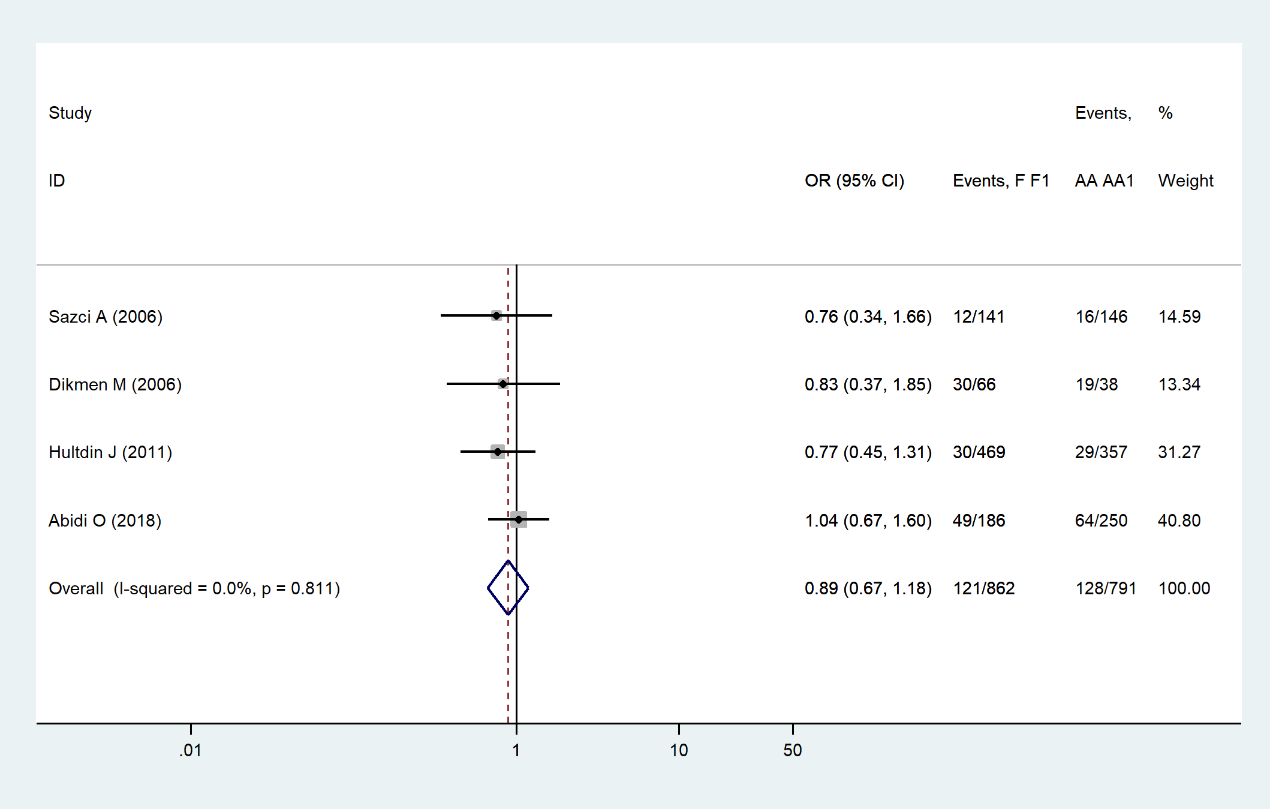


**Supplementary Figure 3(b)**


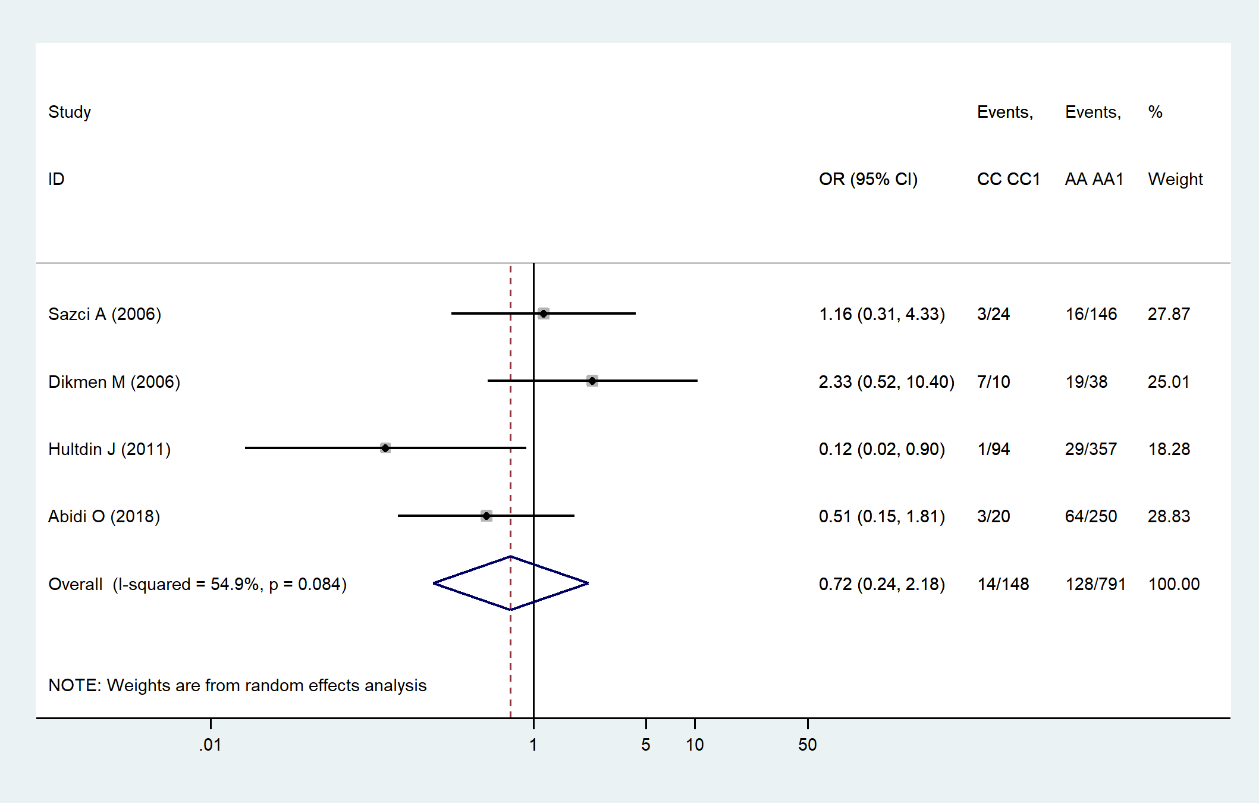


**Supplementary Figure 3(c)**


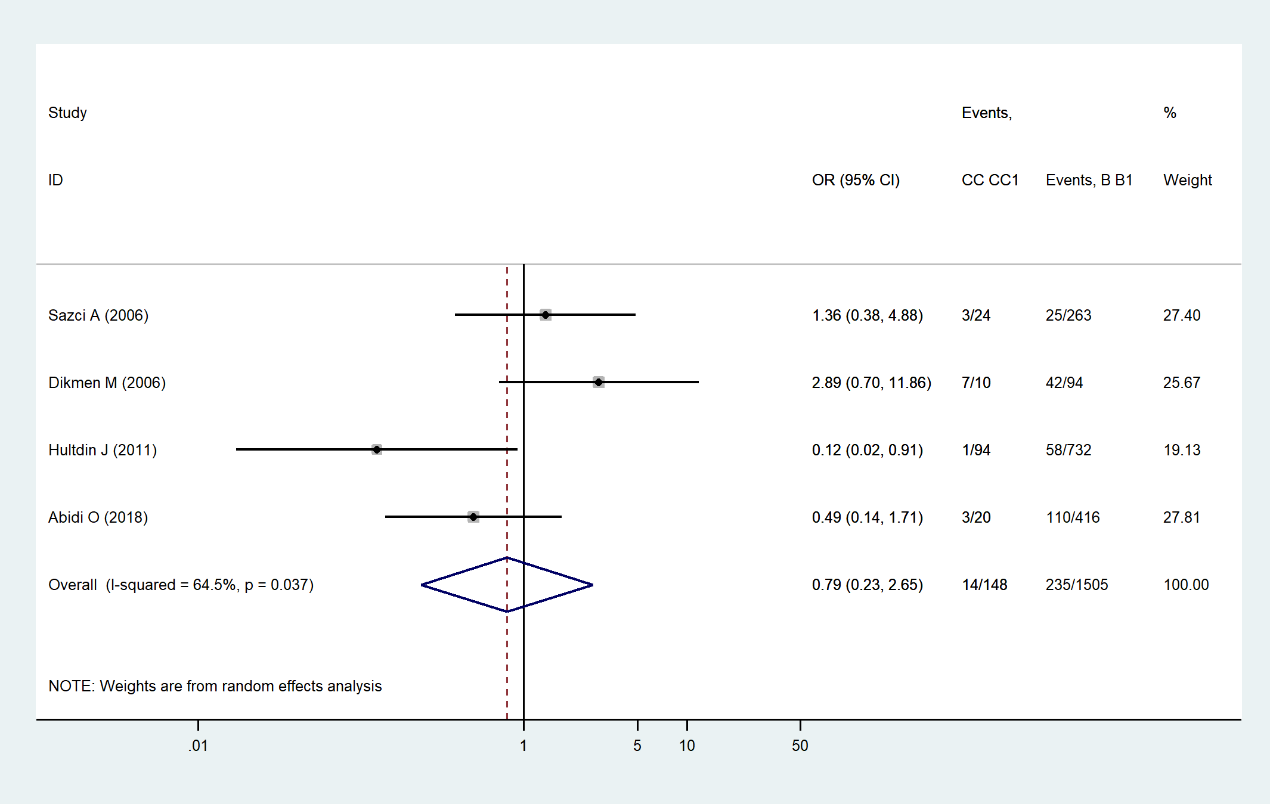


**Supplementary Figure 3(d)**


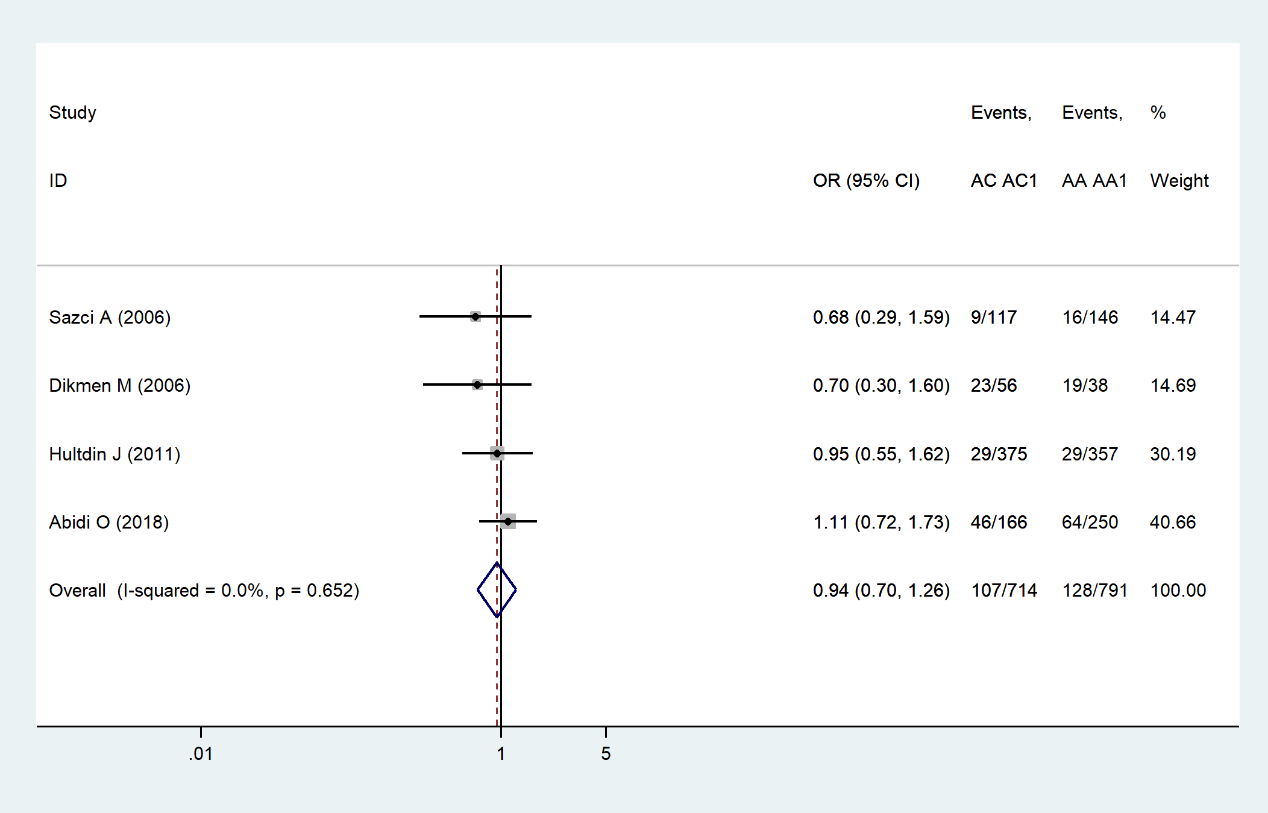


**Supplementary Figure 3(e)**

**Supplementary Figure 3**: **Forest plot of the relationship between MTHFR A1298C gene polymorphism and the risk of ICH.** **a.** Allele model (C VS A), where C and A are case groups and C1 and A1 are control groups. **b.** Homozygous model (CC VS AA), in which CC and AA are case groups and CC1 and AA1 are control groups. **c.** Dominant model (CC+AC VS AA), where F=CC+AC, F1=CC1+AC1.F and AA are case groups, and F1 and AA1 are control groups. **d.** Recessive model (CCVS AA+AC), where B=AA+AC, B1=AA1+AC1.B, AA is the case group, and B1, AA1 are the control group.

**e.** Heterozygous model (AC VS AA), in which AC and AA are case groups and AC1 and AA1 are control groups.


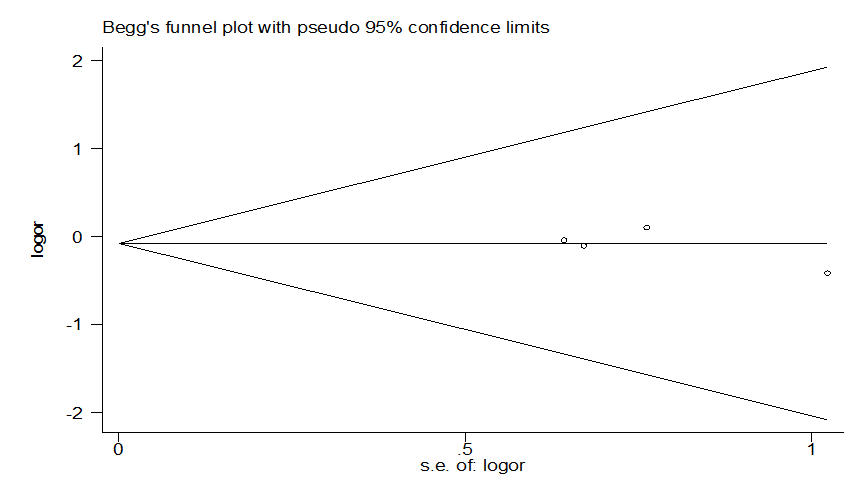


**Supplementary Figure 4: The Begg funnel plot of the relationship between MTHFR A1298C gene polymorphism and the risk of ICH.**
